# Differential regulation of lineage commitment in human and mouse primed pluripotent stem cells by the nucleosome remodelling and deacetylation complex

**DOI:** 10.1016/j.scr.2020.101867

**Published:** 2020-07

**Authors:** Ramy Ragheb, Sarah Gharbi, Julie Cramard, Oluwaseun Ogundele, Susan L. Kloet, Thomas Burgold, Michiel Vermeulen, Nicola Reynolds, Brian Hendrich

**Affiliations:** a Wellcome – MRC Stem Cell Institute, University of Cambridge, Cambridge CB2 0AW, United Kingdom; b Department of Molecular Biology, Faculty of Science, Radboud Institute for Molecular Life Sciences, Radboud University, 6525 GA Nijmegen, The Netherlands; c Oncode Institute, Radboud University, 6525 GA Nijmegen, The Netherlands; d Department of Biochemistry, University of Cambridge, Cambridge CB2 1QR, United Kingdom

**Keywords:** Pluripotency, Chromatin, Lineage commitment, Transcriptomics, iPS cell, epiStem cell

## Abstract

Differentiation of mammalian pluripotent cells involves large-scale changes in transcription and, among the molecules that orchestrate these changes, chromatin remodellers are essential to initiate, establish and maintain a new gene regulatory network. The Nucleosome Remodelling and Deacetylation (NuRD) complex is a highly conserved chromatin remodeller which fine-tunes gene expression in embryonic stem cells. While the function of NuRD in mouse pluripotent cells has been well defined, no study yet has defined NuRD function in human pluripotent cells. Here we find that while NuRD activity is required for lineage commitment from primed pluripotency in both human and mouse cells, the nature of this requirement is surprisingly different. While mouse embryonic stem cells (mESC) and epiblast stem cells (mEpiSC) require NuRD to maintain an appropriate differentiation trajectory as judged by gene expression profiling, human induced pluripotent stem cells (hiPSC) lacking NuRD fail to even initiate these trajectories. Further, while NuRD activity is dispensable for self-renewal of mESCs and mEpiSCs, hiPSCs require NuRD to maintain a stable self-renewing state. These studies reveal that failure to properly fine-tune gene expression and/or to reduce transcriptional noise through the action of a highly conserved chromatin remodeller can have different consequences in human and mouse pluripotent stem cells.

## Graphical abstract

**Figure:**
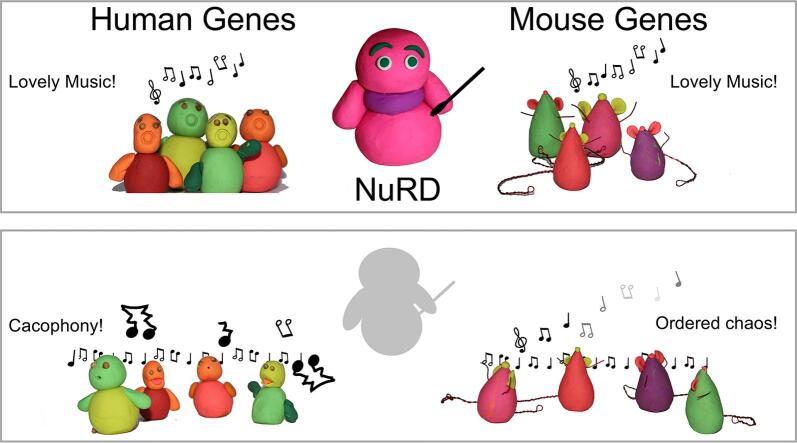

## 1 Introduction

The identity of a eukaryotic cell is ultimately determined by its transcriptional output. The process by which cells transition from one state to another is therefore necessarily subject to tight transcriptional controls. For example, during development, in the absence of changes in external cues, transcriptional programs must remain stable for the identity of that cell to be maintained. Upon changes in external signals, transcription of some genes must be downregulated while that of others must be increased and this results in a change in cellular identity. The mechanisms which act either to maintain or change the expression state of a cell therefore underlie the ordered progression of transitions that occur throughout embryonic development. Failure of regulation of these gene expression patterns can prevent successful execution of developmental decisions, leading to developmental abnormalities, tumorigenesis or death. A comprehensive understanding of how cells control transcription during cell fate decisions is therefore critical for fields where it is desirable to control or instruct cell fate decisions, such as in regenerative medicine or cancer biology.

The ability of cells to activate or repress transcription relies largely on the conformation of the chromatin in which these genes reside. A set of chromatin remodelling complexes function to alter the structure of chromatin at regulatory elements to control gene expression (Hota and Bruneau, 2016). One such complex in particular, the NuRD (**Nu**cleosome **R**emodelling and **D**eacetylation) complex, is important for cells to undergo the changes in identity associated with the exit from pluripotency (Burgold et al., 2019, Kaji et al., 2006, Reynolds et al., 2012a). The NuRD complex is a highly conserved multiprotein chromatin remodeller initially defined as a transcriptional repressor (Tong et al., 1998, Wade et al., 1998, Xue et al., 1998, Zhang et al., 1998). NuRD activity facilitates cell fate transitions in a range of different organisms and developmental contexts (Signolet and Hendrich, 2015). The complex combines two enzymatic activities: class I lysine deacetylation, encoded by the Histone Deacetylase (Hdac) 1 and 2 proteins, with the Swi/Snf-type ATPase and nucleosome remodelling of the Chromodomain Helicase DNA binding protein 4 (Chd4). This complex also contains histone chaperone proteins Rbbp4 and 7, one of the zinc-finger proteins Gatad2a or Gatad2b, two MTA proteins (Mta1, Mta2, and/or Mta3), Cdk2ap1 and Mbd2 or Mbd3 (Allen et al., 2013, Kloet et al., 2015, Mohd-Sarip et al., 2017). Mbd2 and Mbd3 are mutually exclusive within NuRD and while Mbd2/NuRD can confer methyl-CpG binding on a variant NuRD complex (aka MeCP1), MBD2 is dispensable for normal mouse development (Feng and Zhang, 2001, Hendrich et al., 2001, Le Guezennec et al., 2006). In contrast Mbd3 is known to be required for lineage commitment of pluripotent cells and is essential for early mammalian development, demonstrating both that MBD2 cannot functionally substitute for MBD3 and that MBD3/NuRD is the predominant NuRD complex in mammalian cells (Hendrich et al., 2001, Kaji et al., 2006, Kaji et al., 2007). Structural and genetic work has found that Mbd3 physically links two biochemical and functional NuRD subcomplexes: a remodelling subcomplex containing Chd4, the Gatad2 protein and Cdk2ap1; and a histone deacetylase subcomplex containing the Hdac, the Rbbps and the Mta proteins (Burgold et al., 2019, Low et al., 2016, Zhang et al., 2016). Mbd3 thus acts as a molecular bridge between these subcomplexes and maintains the structural integrity of NuRD.

In mouse ESCs (mESCs) Mbd3/NuRD activity modulates the transcription of pluripotency-associated genes, maintaining expression within a range that allows cells to effectively respond to differentiation signals (Bornelöv et al., 2018, Reynolds et al., 2012a). In contrast total NuRD-null mESCs display increased transcriptional noise which exacerbates the lineage commitment defects (Burgold et al., 2019). Despite profound developmental defects, Mbd3 deficiency in mESCs results in only moderate gene expression changes, with the majority of genes changing by less than two-fold. Rather than turning genes on or off, Mbd3/NuRD activity serves to fine-tune gene expression in mESCs (Bornelöv et al., 2018). Although this amounts to many small transcriptional changes, the cumulative effect of this is nevertheless a profound phenotype: the inability of pluripotent cells to undergo lineage commitment. While the function of Mbd3/NuRD in mouse pluripotent cells has been well defined, no study yet has defined NuRD function in human pluripotent cells.

Human and mouse ESCs can both be derived from the inner cell mass (ICM) of pre-implantation epiblasts. Yet the cell lines that emerge after culturing differ in transcriptomic, epigenetic, and morphological features (Nichols and Smith, 2009). mESCs show early developmental characteristics such as the expression of pluripotency genes, DNA hypomethylation and the activity of both X chromosomes in females. Conventional human ESCs (hESCs) are developmentally more advanced and resemble murine post-implantation epiblast or mouse epiblast stem cells (mEpiSCs), and thus are considered to be primed pluripotent (Brons et al., 2007, Tesar et al., 2007). The study of human pluripotent stem cells has been greatly accelerated by the advent of induced pluripotent stem cells (iPSCs), which are derived from somatic cells and thus do not require destruction of a human embryo (Nishikawa et al., 2008, Takahashi and Yamanaka, 2006). As far as can be determined, human iPSCs show a similar level of potency as human ESCs, but also show the range of differentiation biases seen in ESCs, possibly due to differences in culture history, genetic differences between humans, and/or stochastic changes (Osafune et al., 2008, Yamanaka, 2009). For this reason, the differences between two ES cell lines, or two iPS lines is likely to be more significant than any differences between iPS cells and ES cells per se.

In this study we investigated the function of the MBD3/NuRD complex in a human iPSC line, and compared this to its function in mEpiSCs. We find that while MBD3/NuRD is required in both systems for cells to properly undergo lineage commitment, the way in which this function is exerted appears different. Whereas in mouse primed stem cells, as in naïve mESCs, NuRD is required for an appropriate level of transcriptional response to differentiation signals, human primed pluripotent cells require NuRD activity to initiate these transcriptional responses. This difference in the transcriptional consequences upon loss of an orthologous protein in two different mammalian pluripotent stem cell types indicates that mouse and human cells interpret and/or respond to induction of differentiation differently.

## 2 Materials and Methods

### 2.1 Cell lines and culture conditions

hiPSCs were a generous gift of Prof. Austin Smith (Takashima et al., 2014). Endogenously tagged MBD3-3xFLAG hiPSCs were made using a CRISPR/Cas9 gene editing approach to insert 3xFLAG immediately upstream of the MBD3 stop codon using a guide RNA targeting the sequence 5′- GAGCGAGTGTAGCACAGGTG-3′ (Supplemental Fig. 1). *MBD3*-KO cells were generated by replacing exons 2 and 3 with a puromycin resistance cassette using CRISPR/Cas9-mediated targeting and guide RNAs targeting the sequences 5′- GGCGGTGGACCAGCCGCGCC-3′ and 5′- GTCGCTCTTGACCTTGTTGC-3′. A correctly targeted heterozygous clone was then transiently transfected with Dre recombinase prior to a second round of targeting to generate a homozygous null line (Supplemental Fig. 1). The MBD3 Rescue line was made by transfecting the *MBD3*-KO iPS line with a construct containing a CAG promoter driving expression of full-length MBD3-3xFLAG, followed by an IRES and a hygromycin resistance gene, and a polyA sequence from the human *PGK* gene. Hygromycin resistant cells were expanded and tested for MBD3-3xFLAG expression (e.g. Fig. 2A).

hiPSCs were cultured in mTESR1 (StemCell Technologies) media or E8 medium (made in house, prepared according to (Chen et al., 2011)) on vitronectin coated plates. hiPSCs were passaged using an enzyme-free passaging reagent (ReleSR, StemCell Technologies) and plated as small clumps.

Neuroectoderm differentiation was induced based on (Vallier et al., 2009). hiPSCs were plated as clumps (day −1) in chemically defined medium with Polyvinyl Alcohol (CDM-PVA) supplemented with hActivin A (10 ng/µl) and FGF2 (12 ng/µl) on 0.1% gelatin coated plates pre-treated overnight at 37 °C with MEF media (Advanced DMEM-F12, 10% FBS, 2 mM L-glutamine, 1× penicillin/streptomycin). The original composition of CDM is 50% IMDM (Gibco) plus 50% F12 Nutrient-MIX (Gibco), supplemented with 4 µg/ml of insulin (Roche), 15 µg/ml transferrin (Roche), 450 µM monothioglycerol (Sigma), chemically defined lipid concentrate (Invitrogen). The next day (day 0), hiPSCs were cultured in CDM-PVA supplemented with SB431542 (10 µM, Tocris), FGF2 (12 ng/ml, R and D Systems) and Noggin (15 ng/ml, Peprotech) for 12 additional days. The cells were harvested using Accutase at 3, 6 and 12 days. The media was changed every day.

Definitive endoderm differentiation was induced according to (Yiangou et al., 2019). Cells were cultured in CDM-PVA supplemented with 100 ng/ml Activin A (produced in house), 80 ng/ml FGF2 (produced in house), 10 ng/ml BMP4 (R&D Systems), 10 µM LY294002 (Promega) and 3uM CHIR99021 for one day, with CHIR99021 omitted on the second day. From day three onwards, cells were cultured in RPMI basal medium, supplemented with 100 ng/ml Activin A and 80 ng/ml FGF2 on day 3. From day 4 onwards, RPMI was supplemented with 50 ng/ml Activin A only. The cells were harvested using Accutase at days 2, 4, 6 and 8. The media was changed every day.

mEpiSCs were derived from *Mbd3^Flox/Δ^* mESCs and subsequently transiently transfected with Cre recombinase to create *Mbd3*^*Δ/Δ*^ cells. *Mbd3*^*Δ/Δ*^ mEpiSCs were independently derived from *Mbd3*^*Δ/Δ*^ ES cells. mEpiSC cultures were maintained in N2B27 supplemented with FGF2 (12 ng/µl), Activin A (20 ng/µl), XAV939 (2 mM, Sigma) on fibronectin (15 µg/ml) pre-coated plates. The cells were harvested using Accutase at 2, 4 and 8 days. The media was changed every day. For neural differentiation cells were plated on laminin-coated plates in N2B27 containing 1 µM A83-01 (StemMACS).

### 2.2 Gene expression analysis

This was carried out as described (Burgold et al., 2019). Briefly, total RNA was isolated using RNA mini easy kit (Qiagen) and reverse transcribed using random hexamers and Superscript IV Reverse Transcriptase (Invitrogen). Quantitative PCR was carried out using gene-specific primers and Sybrgreen incorporation, or Taqman reagents on a StepOne or ViiA7 real time PCR system (both Applied Biosystems).

Taqman PROBES

**Table:** 

Gene	Probe
β *ACTIN*	Hs01120798_m1
*GATA4*	Hs00171403_m1
*NANOG*	Hs04399610_g1
*NESTIN*	Hs04187831_g1
*PAX6*	Hs00240871_m1
*POU5F1*	Hs04260367_gH
*ZFP42*	Hs01938187_s1

*Nanog*	Mm02019550_s1
*Pax6*	Mm00443081_m1
*Pou5f1*	Mm03053917_g1
*Sox2*	Mm03053810_s1
*Zfp42*	Mm03053975_g1

Primers

**Table:** 

Gene	Forward primer	Reverse primer	Reference
*CRB3*	AGGTCAAAGACGCCCG	TGAAGGCAAAACAGTGCTATTC	
*FOXA2*	GGGAGCGGTGAAGATGGA	TCATGTTGGCTCACGGCGGCGTA	
*FOXG1*	TCACGAAGCACTTGTTGAGG	AGGAGGGCGAGAAGAAGAAC	
*INADL*	GTGATGCCCTTGGAATCAGT	CTGCTCCTCTGTGTCTTCTG	
*SOX2*	GGACAGTTACGCGCACAT	GCTGGTCATGGAGTTGTACT	
*SOX17*	GGCGCAGCAGAATCCAGA	CCACGACTTGCCCAGCAT	
*ZEB1*	CTGACTGTGAAGGTGTACCA	GTACATCCTGCTTCATCTGC	Jiang et al. (2018)

*Ascl1*	CCTCTTAGCCCAGAGGAACA	GTCACTCTTCTCGTGTCTGG	
*Bcam*	GGTGATAGCAAAGGTCCAGG	CCGTTTCGGTACCATGTGAT	
*Ccn2*	ATCTCCACCCGAGTTACCAA	TTTCATGATCTCGCCATCGG	
*Cdh1*	GGCTTCAGTTCCGAGGTCTA	TCTCCAGCTTGTGGAGCTTT	
*Cdkn2a*	GGTTCTTGGTCACTGTGAGG	GTTCGAATCTGCACCGTAGT	
*Dusp4*	GAGGAAAGGGAGGATTTCCA	GTACCTCCCAGCACCAATGA	
*Dusp9*	AGAACGAAGCGGAGGCTA	AATCAGAGCTCAAGCACAGG	
*Epcam*	CCGGGCAGACTCTGATTTAC	CGGCTAGGCATTAAGCTCTC	
*Lefty2*	ACACGCTGGACCTCAAGGAC	GCAGGTCCAGGTACATCTCC	
*Sox3*	TTGCTGTTTAGCTTTGCTCG	TCAACTGCAACAGAAGAACC	

### 2.3 Nuclear extraction, immunoprecipitation and proteomics

Protein extraction and immunoprecipitation were performed as described (Burgold et al., 2019). Original western blot images are available on Mendeley Data: http://dx.doi.org/10.17632/4t99j4c7gx.1. Antibodies used in this study are indicated below:

**Table:** 

Antibody	Raised IN	Company	Catalogue number	Dilution for Western Blot/Immunofluorescence
αCHD4	Mouse	Abcam	ab70469	1:5000
αGATAD2B	Rabbit	Bethyl Labs	A301-281A	1:2000
αLAMIN B1	Rabbit	Abcam	ab133741	1:10,000
αMBD3	Rabbit	Abcam	ab157464	1:5000/1:1000
αMTA2	Mouse	Abcam	ab50209	1:5000
αSOX2	Rat	e-biosciences	14–9811-82	1:500
αβIII Tubulin	Mouse	Cymbus	CBL412	1:1000

Mass spectrometry was carried out as described (Burgold et al., 2019, Kloet et al., 2018, Smits et al., 2013). Briefly, nuclear extract was prepared from a human iPS cell line in which a 3xFLAG tag was knocked in to the endogenous *MBD3* locus, or from two independent mouse epiStem cell lines similarly modified as described (Burgold et al., 2019). One preparation of nuclear extract from each cell line was divided into thirds, which were independently processed for proteomic analyses. Proteins associated with 3xFLAG-tagged MBD3 were purified using anti-FLAG sepharose (Sigma) and processed for mass spectrometry as described (Smits et al., 2013). The resulting data were processed as in (Kloet et al., 2018).

### 2.4 RNA-seq and analysis

Sequencing libraries were prepared using the NEXTflex Rapid Directional RNA-seq kit (Illumina) or SMARTer® Stranded Total RNA-Seq Kit v2—Pico Input Mammalian (Takara Bio) and sequenced on the Illumina platform at the CRUK Cambridge Institute Genomics Core facility (Cambridge, UK). Illumina sequence files were converted into FASTQ format. The short sequence reads (75 nucleotides) were aligned to the Human reference genome (hg38; http://genome.ucsc.edu/) or to the Mouse reference genome (mm10; http://genome.ucsc.edu/) and assigned to genes using BWA (Li and Durbin, 2009). We used the Subread package (R statistical tool; http://www.r-project.org/) to count aligned reads. Differentially expressed genes were identified using R package edgeR (Chen et al., 2016). We used no fold change filtering and results were corrected for multi-testing by the method of the False Discovery Rate (FDR) at the 1% level. Differentially expressed genes were clustered using the unsupervised classification method of the Kmeans (Soukas et al., 2000). Heat maps were created using the pheatmap function (R statistical tool; http://www.r-project.org/). Functional annotation enrichment for Gene Ontology (GO) terms was determined using the HumanMine [http://www.humanmine.org] (Smith et al., 2012)or MouseMine databases [http://www.mousemine.org]. Benjamini-Hochberg corrected P values of less than 0.01 were considered significant. GO terms were submitted to REVIGO, a web server that takes long lists of GO terms and summarizes them in categories and clusters of differentially expressed genes by removing redundant entries (Supek et al., 2011). We used the i-*cis*Target tool (Imrichova et al., 2015) to look for enrichment in TF position weight matrices and potential binding sites in the regulatory regions of co-expressed genes. i-cisTargetX computes statistical over-representation of DNA motifs and ChIP-seq peaks in the non-coding DNA around sets of genes. The enrichment was considered significant when the Normalized Enrichment Score (NES) was higher than 5.

### 2.5 Data availability

RNA-seq data are available with the Array Express accession number E-MTAB-8753. The mass spectrometry proteomics data have been deposited to the ProteomeXchange Consortium via the PRIDE partner repository with the dataset identifier PXD016967. All original western blot images are available at Mendeley Data: DOI: https://doi.org//10.17632/4t99j4c7gx.1.

## 3 Results

### 3.1 NuRD complex structure is conserved in mouse and human pluripotent stem cells

**Fig. 1:**
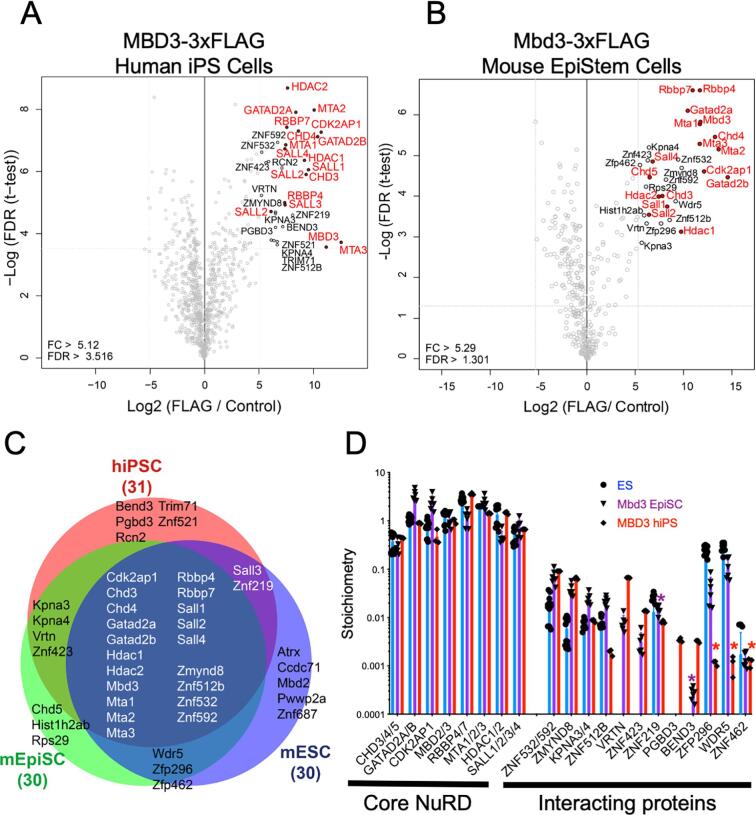
Comparison of human and mouse NuRD complexes. A) and B). Proteins associated with MBD3/NuRD in hiPSCs (A) or mEpiSCs (B) were identified by immunoprecipitation and mass spectrometry. Proteins significantly associating with MBD3 are indicated, with NuRD component proteins indicated in red. The human data comprise three independent immunoprecipitations from one preparation of nuclear extract, while the mouse data comprise three independent immunoprecipitations each from nuclear extract preparations made from two independent cell lines. C) Overlap of proteins significantly associating with MBD3 in hiPSCs, Mbd3 in mEpiSCs, and Mta1, 2 or 3 in mouse ESCs (taken from (Burgold et al., 2019)) is displayed. The numbers in parentheses indicate the number of significantly enriched proteins in each experiment. D) Relative enrichment of indicated proteins normalised to the bait protein for each experiment: for mouse, ESC data (a combination of mass spectrometry experiments using Mta1, Mta2 and Mta3 as bait, taken from (Burgold et al., 2019); blue) were normalised to two MTA proteins, while for both the EpiSC (purple) and hiPSC (red) experiments the data were normalised to one MBD3 protein. Error bars represent standard deviation of three (hiPSCs), six (mEpiSCs) or nine (mESCs) replicates. Asterisks indicate situations where the protein enrichment was not significant in this cell type, but the stoichiometry is displayed for comparison. (For interpretation of the references to colour in this figure legend, the reader is referred to the web version of this article.)

In order to characterise human NuRD, we used genome editing to insert coding sequence for a 3xFLAG epitope immediately upstream of the stop codon of one endogenous *MBD3* allele in human iPS cells (Fig. S1A, B). An equivalent C-terminally tagged murine endogenous MBD3 protein shows genomic localisation identical to that found for wild type MBD3 protein in mouse ES cells, and supports normal embryonic development in mice (Bornelöv et al., 2018). Biochemical isolation of MBD3/NuRD in MBD3-3xFLAG hiPSCs, or in mEpiSCs containing an identically modified *Mbd3* allele, followed by mass spectrometry identified all known components of NuRD in both systems (Fig. 1A, B). A number of interacting proteins were also purified at much lower stoichiometries than was seen for core NuRD components. Comparison of mass spectrometry data between hiPSCs, mEpiSCs and mouse naïve ES cells (using MTA1-3 proteins for NuRD purification: (Burgold et al., 2019)) showed that most interacting proteins identified in human cells also interact with mouse NuRD (Fig. 1C). Two cell-type specific interactors are VRTN and ZNF423, both of which are not expressed in naïve ES cells, but are found interacting with NuRD in primed PSCs (mEpiSCs and hiPSCs; Fig. 1C). Two nuclear proteins were identified interacting with human NuRD that were not significantly enriched in the mouse datasets: PGBD3 and BEND3. PGBD3 is a transposase - derived protein expressed as a fusion with ERCC6 not present in mice (Newman et al., 2008), but previously reported to interact with NuRD components in human cells (Hein et al., 2015). Although not significantly detected in our mouse NuRD purifications, BEND3 has previously been shown to recruit NuRD to major satellite repeats in mouse cells (Saksouk et al., 2014). WDR5, ZNF296 and ZNF462 were identified interacting with mouse NuRD as described (Burgold et al., 2019, Ee et al., 2017, Kloet et al., 2018), but were beneath our significance cut off in purifications from human cells (Fig. 1C). We therefore conclude that NuRD structure and biochemical interactors are generally conserved between mouse and human PSCs.

**Fig. 2:**
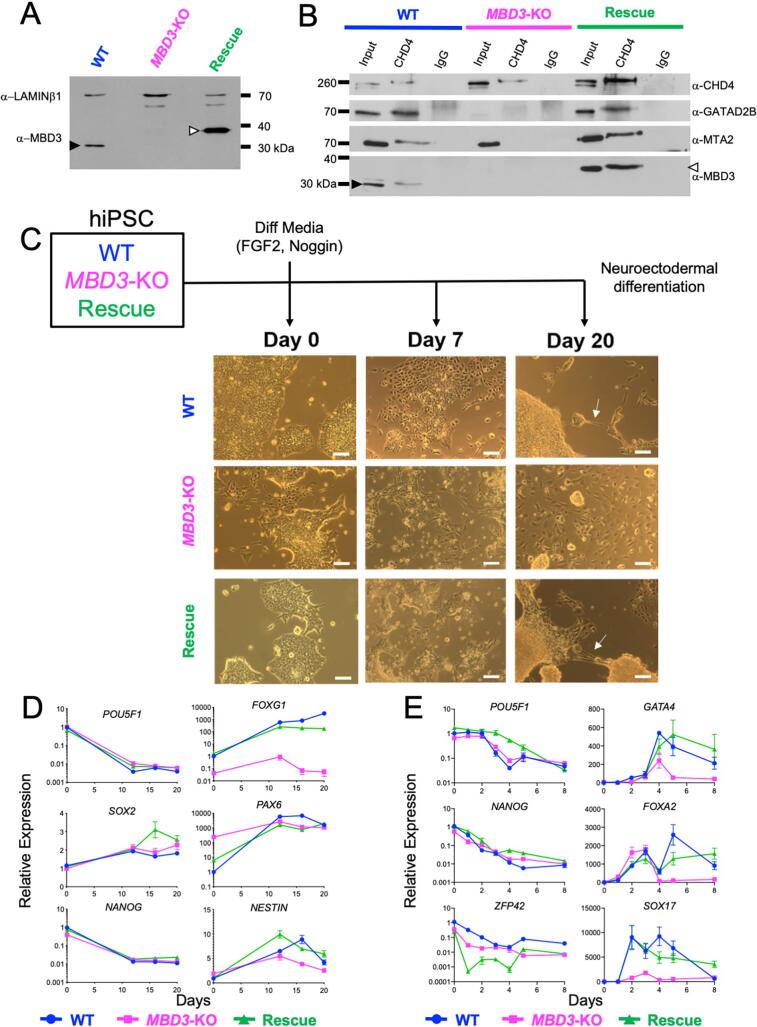
Human iPS cells lacking MBD3/NuRD fail to undergo programmed differentiation. A) Western blot of nuclear extracts from wild type (WT), *MBD3*-KO and *MBD3*-KO hiPSCs rescued with an MBD3-3xFLAG transgene (“Rescue”). The blot was probed with antibodies indicated at left. The closed arrowhead indicates native MBD3, while the open arrowhead indicates the MBD3-3xFLAG fusion protein. B) Nuclear extracts from wild type, *MBD3*-KO and Rescued cells was immunoprecipitated with anti-Chd4, western blotted and probed with antibodies indicated at right. Arrowheads as in Panel A. C) Scheme of the differentiation experiment (top) and images of indicated cell cultures at Day 0, 7 or 20 of differentiation. Scale bar indicates 100 µm. D) Expression of pluripotency (*POU5F1, SOX2* and *NANOG*) and lineage specific genes (*FOXG1, PAX6* and *NESTIN*) during neural differentiation was measured by qRT-PCR. Y-axis represents expression relative to that in wild type cells at Day 0, while the X-axis represents the time in days. Error bars represent the standard deviation of ≥3 biological replicates. E) as in panel D, but for definitive endoderm differentiation protocol. Pluripotency-associated genes (*POU5F1*, *NANOG*, *ZFP42*) on the left, and differentiation-associated genes (*GATA4*, *FOXA2*, *SOX17*) at right.

### 3.2 NuRD mutant hiPS cells are unable to maintain a stable pluripotency state

We next asked what function was played by MBD3/NuRD in human PSCs. To this end we used CRISPR/Cas9-mediated gene targeting to create an *MBD3*-KO iPSC line (Fig. 2A; Fig S1C). Immunoprecipitation with the remodelling subunit of NuRD, CHD4, allowed for purification of other complex components in wild type cells, but not in the *MBD3*-KO cells, indicating that human NuRD does not form without MBD3 (Fig. 2B). Interactions between CHD4 and other NuRD components were restored when an MBD3 transgene was overexpressed in the null cells (“Rescue”, Fig. 2A,B), indicating that the transgenic MBD3 is sufficient for NuRD formation as it is in mESCs (Bornelöv et al., 2018, Reynolds et al., 2012b).

*MBD3*-KO hiPSCs were viable in standard culture conditions (mTESR or E8 (Chen et al., 2011)), though unlike wild type cells null cultures showed some degree of spontaneous differentiation in both culture conditions (Fig. 2C). While wild type and Rescue cultures presented as morphologically homogeneous colonies with clear boundaries, mutant cultures were a mix of cells showing a compact, undifferentiated morphology as well as a population of flatter, less dense colonies with irregular boundaries, reminiscent of differentiated cells (Fig. 2C). This was surprising since *Mbd3*-KO mESCs are resistant to differentiation (Kaji et al., 2006).

Wild type, mutant and Rescued hiPSCs were induced to differentiate towards a neuroectodermal fate or a definitive endoderm fate to determine whether NuRD was required for successful lineage commitment of human pluripotent cells (see Methods). After 20 days of neuroectodermal differentiation, axon-like extensions were readily identifiable in wild type and Rescue cultures (Fig. 2C). In contrast, no such appendages were found in *MBD3*-KO cultures, indicating a requirement for MBD3/NuRD for successful completion of this differentiation process. All three cell lines showed a decrease in expression of pluripotency markers across both differentiation protocols, indicating that NuRD is not required for PSC to respond to differentiation signals (Fig. 2D, E). *MBD3*-KO cells failed to properly induce expression of some lineage-appropriate genes in both differentiation protocols, but this ability was restored upon rescue with the MBD3 transgene (Fig. 2D, E). While NuRD is therefore required in human cells to faithfully maintain a self-renewing state, it is also required for appropriate lineage determination in these two differentiation protocols.

**Fig. 3:**
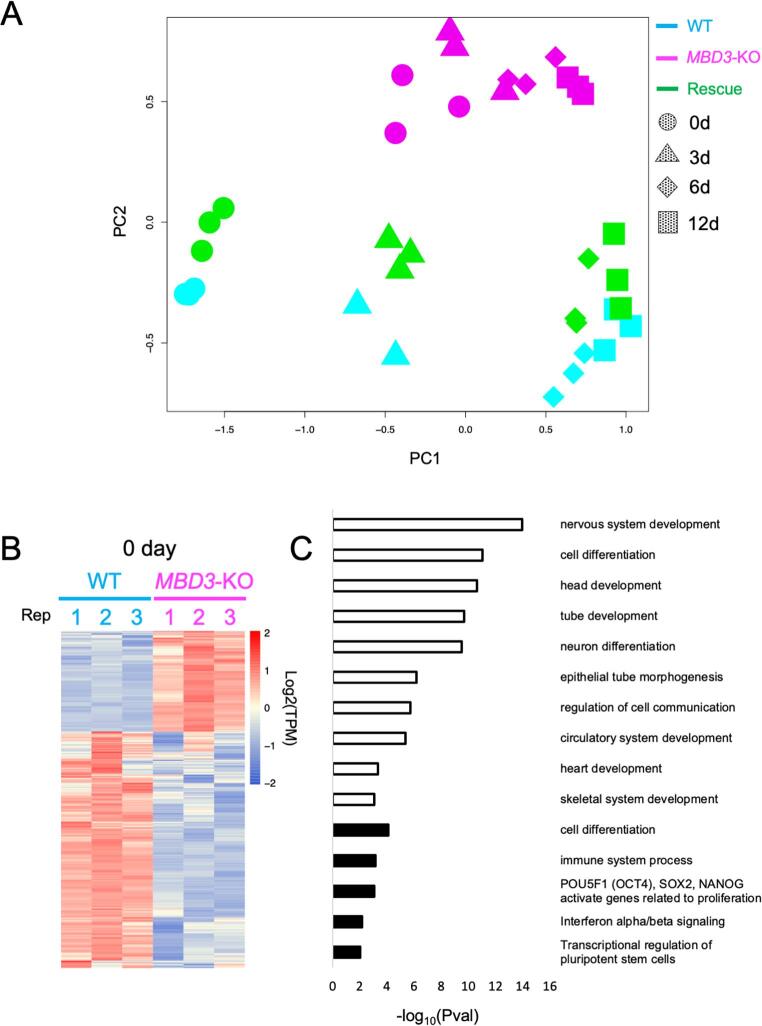
NuRD is required to maintain a stable pluripotency state in hiPSCs A) MDS plot made from RNA-seq data of wild type, *MBD3*-KO and Rescued hiPSCs across a neural differentiation timecourse. Each point represents a biological replicate, and shapes indicate the days of differentiation. B) Heat map of genes found to be differentially expressed between WT and *MBD3*-KO cells in self-renewing conditions (day 0; FDR 5%). C) GO terms associated with genes significantly activated (open bars) or repressed (filled bars) in *MBD3*-KO cells relative to WT hiPSCs in self-renewing conditions.

To determine how NuRD facilitates lineage commitment in hiPSCs, we analysed and compared the transcriptomes of WT, *MBD3*-KO and Rescue cells at 0, 3, 6 and 12 days upon neuroectodermal differentiation. Visualising the data using a multidimensional scaling plot (Ritchie et al., 2015) separated each sample along the time of differentiation, represented by PC1, and the genotype, represented by PC2 (Fig. 3A). Data from WT and Rescue cells clustered close to each other and followed a similar developmental trajectory, indicating that overexpression of *MBD3* does not dramatically impair early stages of neural differentiation. In contrast, data from *MBD3*-KO cells clustered separately, indicating that they are undergoing aberrant differentiation, consistent with our RT-qPCR data (Fig. 2D). At day 0 NuRD mutant cells occupy a position further along the differentiation trajectory (PC1) than do either WT or Rescue cells, likely resulting from the presence of morphologically differentiated cells within the self-renewing *MBD3*-KO cultures (Fig. 2C).

To try to understand why *MBD3*-KO PSC were unable to stably maintain an undifferentiated state, the transcriptomes of WT and *MBD3*-KO PSCs were compared in self-renewing conditions (Fig. 3B). Null cells showed 823 differentially expressed genes compared to wild type cells (246 up- and 577 down-regulated, FDR 1%). GO term enrichments performed on the set of down-regulated genes showed terms related to pluripotency (Fig. 3C, Table S1) consistent with a failure to maintain a stable pluripotent state in the absence of MBD3/NuRD. Expression profiles of pluripotency markers (*NANOG*, *TDGF1*, *FOXD3* and *FGF2*) showed a significant down-regulation in mutant cultures when compared to the WT or Rescue cells (Table S1). The 246 up-regulated genes showed enrichment for terms related to the development of different lineages (Fig. 3C, Table S1). Given that *MBD3*-KO cells failed to undergo programmed neural or endodermal differentiation despite precociously expressing differentiation markers, we conclude that, like in mESCs (Burgold et al., 2019), human NuRD functions to prevent inappropriate gene expression in undifferentiated pluripotent cells, and this noise reduction function is important for faithful execution of lineage decisions.

### 3.3 Human NuRD activity is required for appropriate transcriptional response to differentiation signals

**Fig. 4:**
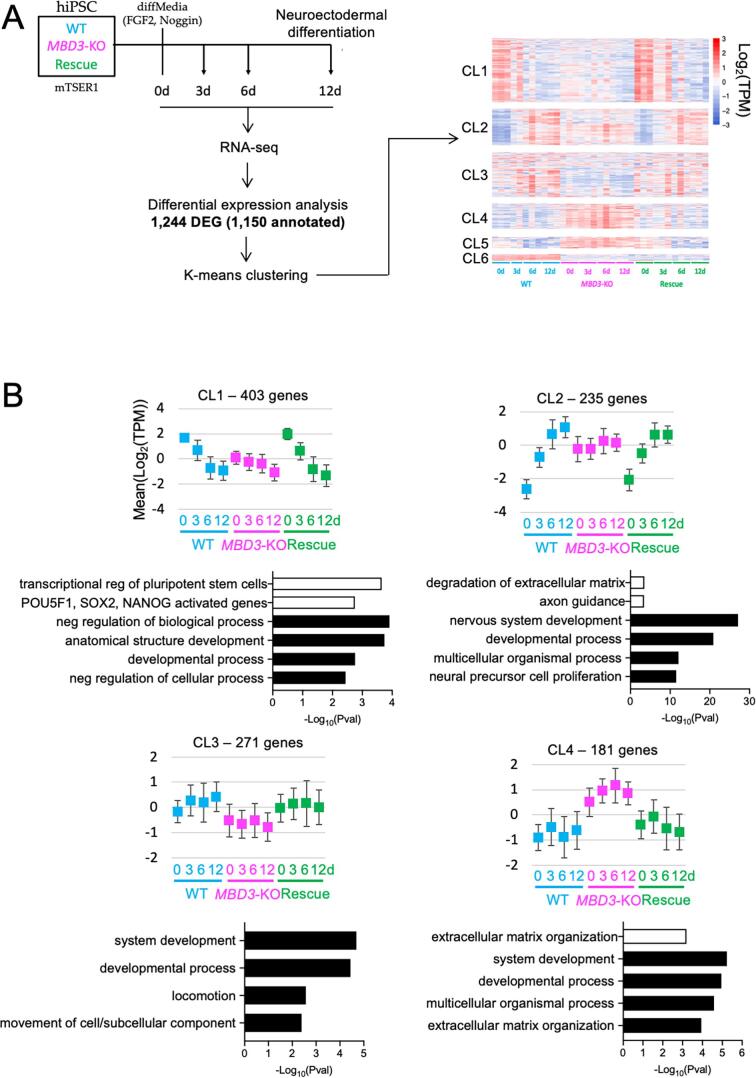
NuRD is required for transcriptional responsiveness in hiPSCs. A) Scheme of the experiment. Wild type (WT), *MBD3*-KO or Rescued cells maintained in mTESR1 media were subjected to neural differentiation. Cultures were sampled at indicated time points for RNA-seq, leading to the identification of 1150 annotated differentially expressed genes (DEG; FDR 1%). Kmeans clustering of genes by expression pattern led to the heat map shown at right, with six major gene clusters. B) Mean expression for genes in each cluster is displayed across the differentiation time course for each cell line. Error bars indicate standard deviations of average expression. The four most significant GO terms associated with each cluster are plotted as solid bars, while up to two pathways (P.adj ≤ 0.01) are also plotted in open bars. A full list of GO terms and pathways is available in Tables S4 and S5.

To better understand how human NuRD facilitates lineage commitment, we next asked how gene expression changes during the differentiation time course differed in *MBD3*-KO cells as compared to WT or Rescue cells. By considering both genotype and the time of differentiation in our differential analysis, we identified genes showing expression changes in at least one cell line compared to the others during differentiation. Clusters of co-expressed genes were identified using K-means clustering (Li et al., 2018), resulting in 6 groups showing similar expression profiles (Fig. 4A).

Cluster 1 is composed of 403 genes down-regulated during normal differentiation (Fig. 4A, B; Tables S2–S4). These genes were generally underexpressed in *MBD3*-KO hiPSCs, yet become further down-regulated as cells are subjected to differentiation conditions. This cluster includes pluripotency-associated genes such as *POU5F1*, *NANOG*, *FOXD3*, *TDGF1*, *FGF2*, *ZSCAN10*, *DPPA4* and *PRDM14*, validating and extending our conclusion drawn from data shown in Fig. 3 that *MBD3*-KO hiPSCs have a defect in maintaining the self-renewing state in standard conditions. Transcription factor binding sites enriched within 10 Kb of the TSS of genes in this cluster showed significant enrichment of consensus binding sites for general transcription factors associated with activation of pluripotency gene expression (i.e. MYC, ATF, CEBPB; Table 1), consistent with a decrease in expression of pluripotency-associated genes. This analysis additionally identified consensus binding sites for SNAIL and ZEB1 (Table 1), transcription factors associated with epithelial to mesenchymal transition as well as repression of pluripotency gene expression (Jiang et al., 2018, Moreno-Bueno et al., 2008). Human ES cells undergo EMT as part of the differentiation process (Kim et al., 2014), so this likely results from inappropriate expression of epithelial genes which would normally precede an EMT event.

**Table 1:** Transcription factor binding sites associated with gene clusters.

Human cluster	TF	NES score
Cluster 1	MYC	7.99
	ATF4	7.27
	CEBPB	6.57
	SNAI2	5.85
	ZEB1	5.25
Cluster 2	NA	NA
Cluster 3	SRF	6.06
Cluster 4	NA	NA
Cluster 5	NA	NA
Cluster 6	TP53	*8.07*
	GFI1B	*6.23*

**Mouse cluster**	**TF**	**NES score**
Cluster 1	Zeb1	6.62
	Rela	5.85
	Smarcc1	5.74
	Fos/Jun	5.57
	Klf1	5.21
	Tead3	5.12
Cluster 2	NA	NA
Cluster 3	NA	NA
Cluster 4	Trp73	5.05

**Overlap cluster**	**TF**	**NES score**
Cluster 1	RBBP9	5.33
	SF1	5.01
Cluster 2	ZEB1	5.54
	SNAI2	5.43
	RNF114	5.25
	PPARG	5.10
Cluster 3	PSMA6	5.25
	SOX6	5.04

Genes in clusters 2–4 are predominantly associated with GO terms involved in differentiation (Fig. 4B; Tables S2–S4). Cluster 2 contains genes associated with neuroectodermal differentiation and were induced in wild type and Rescue cells. Cluster 2 genes (including *PAX6*, *OTX1* and *SOX1)* showed inappropriate expression in *MBD3*-KO cells at time 0 and remained high throughout the differentiation time course. Cluster 3 genes, some of which are associated with neuronal maturation (such as *PLP1*, *SEMA3A*, and *APP*) were expressed at a lower level in mutant cells than in either WT or Rescue cells. Cluster 4 contains genes not induced in either WT or Rescue cells, but which showed inappropriate expression in *MBD3*-KO cells at all time points (e.g. *WNT5A*, *FOXA2*, *PAX7*; Fig. 4B, Table S2). Cluster 5 contains only 27 genes which fail to be appropriately silenced during differentiation in *MBD3*-KO cells, but show no significant enrichment with any GO term, and genes in Cluster 6 show similar expression patterns in mutant and Rescue cells, and are hence unlikely to contribute to the differentiation failure phenotype of *MBD3*-KO cells (Fig. S2).

Genes in Clusters 2 and 4 are not associated with any specific TF binding sites, while Cluster 3 genes show enrichment of binding sites for the general transcription factor SRF (Table 1). This lack of evidence for misregulation of a specific transcriptional programme indicates that *MBD3*-KO hiPSCs fail to interpret a range of different differentiation signals, as opposed to just one or two main pathways. The silencing of pluripotency-associated genes (Cluster 1) in *MBD3*-KO cells demonstrates an ability of these cells to respond to the loss of self-renewal signals, yet the failure of genes in Clusters 2 and 5 to appropriately change expression during the differentiation time course indicates that NuRD is required for cells to properly respond to differentiation signals.

### 3.4 MBD3/NuRD controls lineage commitment differently in human and mouse primed PSC

**Fig. 5:**
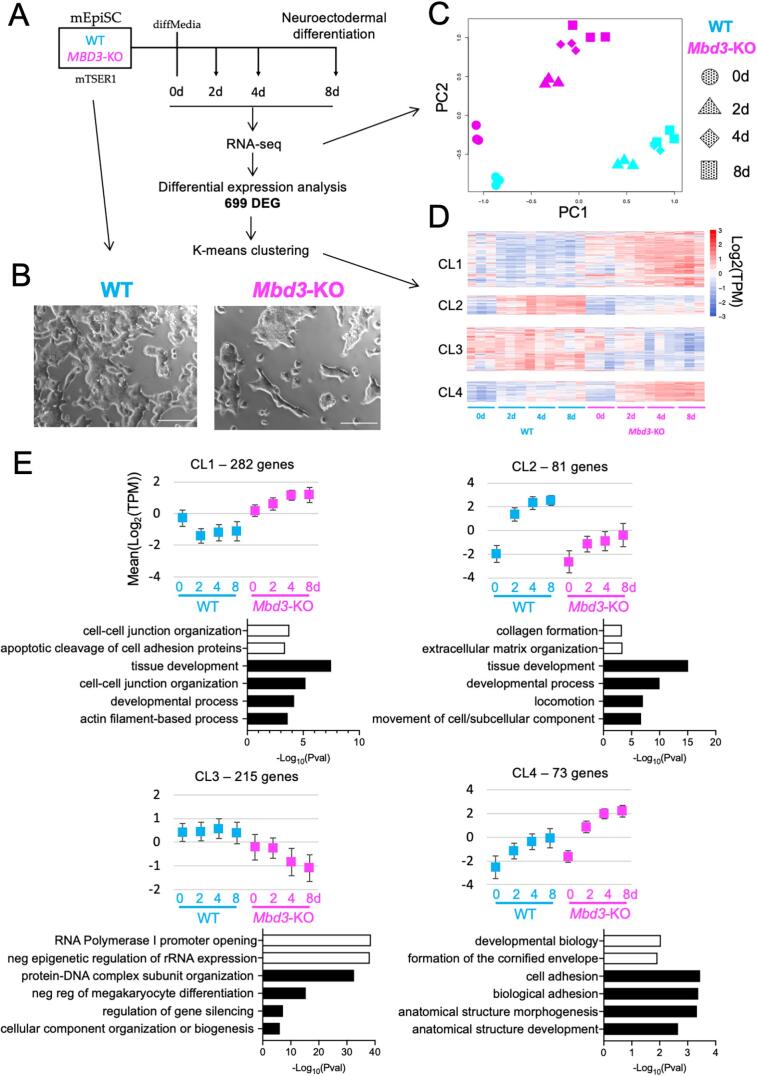
NuRD facilitates an appropriate transcriptional response in mEpiSCs. A) An outline of the experiment, as in Fig. 4. B) Phase contrast images of wild type or *Mbd3*-KO mEpiSCs in self-renewing conditions. C) MDS plot of gene expression data collected across the neural differentiation time course, as in Fig. 3A. D) Heat map of DEG (FDR 1%) separated into four clusters by K-means clustering. E) Mean expression and most significant GO terms for each cluster as in Fig. 4B. A full list of GO terms and pathways is available in Tables S6 and S7. Gene expression changes during neural differentiation of an independent pair of WT and Mbd3-KO EpiSCs, verifying the results of the RNA-seq shown here, is displayed in Fig. S4.

Mbd3/NuRD facilitates exit from the self-renewing state in mouse naïve ESCs (Burgold et al., 2019, Kaji et al., 2006) and thus it was surprising that human primed PSC required MBD3/NuRD to properly maintain the self-renewing state. To determine whether this was a difference between human and mouse cells, or between naïve and primed pluripotent cells we next asked whether NuRD was required to maintain the self-renewing state in mEpiSCs. *Mbd3^−/−^* mEpiSCs were derived in culture from *Mbd3^−/−^* mESCs or by transient Cre expression in mEpiSCs derived from *Mbd3^Flox/-^* mESCs (See Methods). *Mbd3^−/−^* mEpiSCs derived through either method were indistinguishable, and appeared uniformly undifferentiated (Fig. 5A, B), indicating that the spontaneous differentiation seen in hiPSCs does not reflect a general requirement for MBD3/NuRD to maintain primed PSC.

To further compare mouse and human primed PSC, gene expression was monitored across a neural differentiation time course in two independent pairs of Floxed and *Mbd3^−/−^* mEpiSCs by RNA-seq (Fig. 5A, S3; Tables S5–S7) and RT-qPCR (Fig. S4). When the data are visualised using a multidimensional scaling plot (Chen et al., 2016), each sample separates along PC1, representing time of differentiation, and PC2, representing genotype (Fig. 5C). Control and *Mbd3^−/−^* cells occupy the same position along the differentiation trajectory (PC1), consistent with our observation that mEpiSCs do not require MBD3/NuRD to maintain a morphologically undifferentiated state.

As with the hiPSCs, we considered both the genotype and the time of differentiation in our analysis and thus identified 699 differentially expressed genes. Co-expressed genes were grouped using K-means clustering, resulting in 4 clusters showing similar expression profiles (Fig. 5D). In contrast to the human cells which generally showed a lack of transcriptional response to the differentiation time course, clusters identified in mouse cells showed transcriptional responses, but these responses differed from those in wild type cells (Fig. 5E). Cluster 1 genes showed decreased expression in wild type cells across the time course, but increased expression in *Mbd3^−/−^* cells. Genes in clusters 2 and 4 also show increases in both wild type and mutant cells, but Cluster 2 genes showed a reduced response in mutant cells, whereas cluster 4 genes showed an increased response. Cluster 3 genes decreased in expression in mutant cells across the time course, but showed no overall change in wild type cells. In all clusters the genes are associated with very general GO terms, and are unlikely to represent individual pathways or developmental trajectories (Fig. 5E; Tables S6 and S7). These data indicate that in mEpiSC Mbd3/NuRD is not strictly required for cells to respond to differentiation signals as was seen in hPSC, but rather is required for an appropriate level of response, as it is in naïve mESC (Burgold et al., 2019). Rather than being required for the transcriptional response to differentiation cues as in the human differentiation course, Mbd3/NuRD functions in mouse primed PSC to facilitate an appropriate transcriptional response to neural induction.

**Fig. 6:**
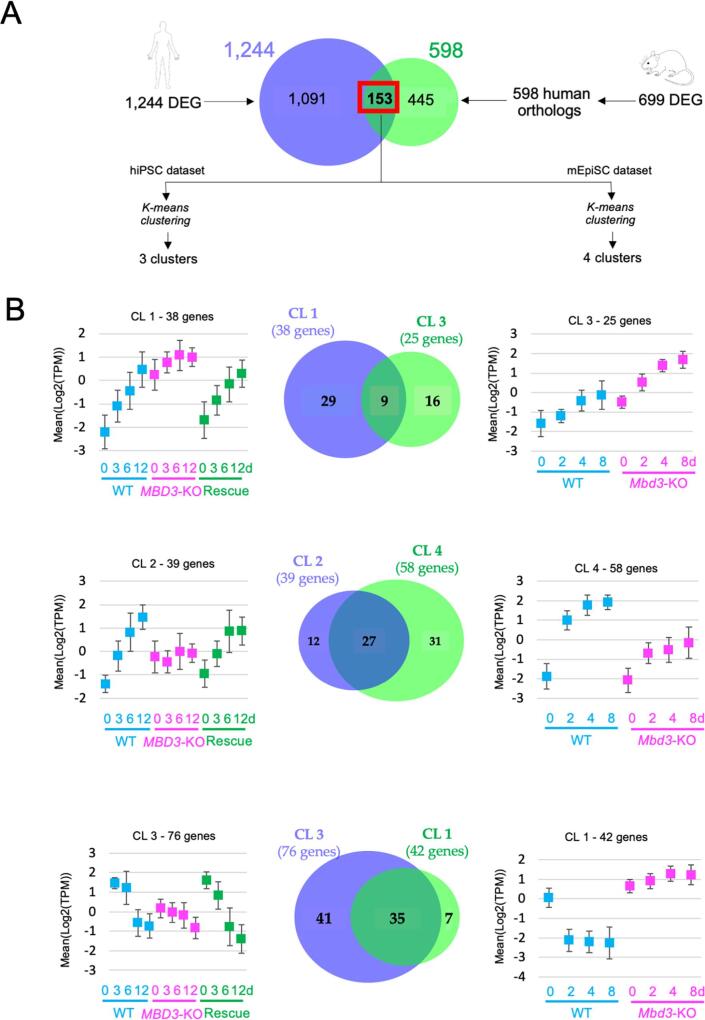
MBD3/NuRD deficiency elicits a different response in human and mouse primed pluripotent stem cells. A) Schematic of the analysis: the Venn diagram shows the overlap of differentially expressed genes identified in human cells, and the identified human orthologues of those identified in mEpiSCs. K-means clustering of this set of 153 genes in human data led to the formation of three gene clusters and to four gene clusters in mouse data. B). Expression profiles are shown for the different gene clusters. Error bars indicate standard deviations of average expression. Expression profiles of human and mouse gene clusters with similar expression patterns in wild type cells are displayed together, and the number of common genes is shown. GO terms are available in Table S9.

Despite the differences in NuRD-dependent gene regulation observed in mouse and human primed PSC described thus far, we asked whether there could be a conserved core set of genes regulated similarly in primed PSC from both species, which might contribute to the shared requirement for NuRD in lineage commitment. Comparing gene expression datasets between the human and mouse experiments (Fig. 6A) identified 153 genes, the orthologues of which were differentially expressed in both human and mouse cells. K-means clustering of data from human and mouse cells separately segregated the genes into three or four main clusters respectively (Fig. 6; Table S8). We next assessed the impact of *MBD3* mutation on the behaviour of clusters which showed similar expression profiles in WT cells (Fig. 6; Table S9). Human cluster 1 and mouse cluster 3 were both induced in wild type cells across the differentiation time course, and were overexpressed in both cell types at time 0, but in mouse cells these genes showed some degree of further up-regulation across the time course, whereas there was no significant increase in expression of these genes in human cells (Fig. 6B, *top*). These clusters have only 9 genes in common and return no significant GO terms (Table S9). The two other comparisons feature genes associated with general developmental GO terms (Table S9) that are activated during differentiation (human cluster 2/mouse cluster 4), or genes which are silenced upon normal differentiation and are associated with epithelial development and cell–cell contacts (human cluster 3/mouse cluster 1; Table S9). In both cases the behaviour of cluster genes in MBD3-null cells differs in the two species: while human cluster 2 genes fail to be activated in MBD3-null cells, mouse cluster 4 genes do increase in expression, but to a much lesser extent than in wild type cells. Human cluster 3 genes are underexpressed in MBD3-null cells and remain low throughout the differentiation time course, while mouse cluster 1 genes are expressed at inappropriately high levels in Mbd3-null mouse cells and remain high during differentiation (Fig. 6). If, instead of clustering human and mouse genes separately, we ask whether mouse genes orthologous to those in the human clusters behave similarly, we get a similar picture: in general mouse genes do not behave the same as the human genes (Fig. S6; Tables S9 and S10). We therefore conclude that the transcriptional consequences of Mbd3/NuRD loss are different in human versus mouse primed PSC, but in both cases this activity is required for cells to properly undergo lineage commitment.

## 4 Discussion

Differentiation of mammalian pluripotent cells involves large-scale changes in transcription, which result in loss of one cell identity and gain of a new, more differentiated identity. Orchestrating these changes in transcription are a large cast of different transcription factors and signalling molecules, but there is also a set of chromatin remodellers whose activity is essential to initiate, establish and maintain a new gene regulatory network (GRN) (Gokbuget and Blelloch, 2019, Hota and Bruneau, 2016). When induced to differentiate, both mouse and human pluripotent stem cells depend on NuRD activity to elicit an appropriate transcriptional response and undergo lineage commitment, but the manner in which NuRD is used to facilitate this response differs. Induction to differentiate elicits changes in transcription from a range of genes in both human and mouse PSC, though the identity of the specific genes largely differs in the two species (Fig. 6A). The absence of MBD3/NuRD activity in human cells results in a subset of these genes failing to respond to the differentiation cues, while in mutant mouse PSC the response is present and widespread, but often muted or inappropriate (Fig. 4, Fig. 5). This subtle difference in NuRD-dependent gene expression changes could conceivably give rise to quite different downstreatm consequences, and may underlie the rather low percentage of genes commonly misregulated during neural differentiation of mouse or human PSC (Fig. 6A). NuRD activity is additionally required to maintain the pluripotency GRN of hiPSCs cultured in self-renewing conditions, but neither primed nor naïve mouse PSCs display this requirement (Fig. S3A and (Kaji et al., 2006)). We see no large-scale differences in the biochemical make-up of NuRD between human and mouse primed stem cells, which would be consistent with the human and mouse complexes exerting similar, or identical biochemical functions. The observed differences in the consequences of MBD3 deficiency are therefore likely to result from subtle differences in how NuRD activity is used by the cells to respond to changes in environment.

One example of how human and mouse cells respond differently to loss of MBD3/NuRD is in regulation of the *ZEB1/Zeb1* genes. *ZEB1* is overexpressed in self-renewing human *MBD3*^*-/-*^ PSC and remains high through the neural induction time course, whereas in mouse *Mbd3*^*-/-*^ cells *Zeb1* is underexpressed in self-renewing cells and fails to be activated during the time course (Fig. S5). ZEB1 has been shown to repress polarity and gap-junction genes associated with an epithelial morphology, promoting an epithelial to mesenchymal transition (EMT) (Aigner et al., 2007). This function is required for neural differentiation in vivo and from hESCs in culture (Jiang et al., 2018, Singh et al., 2016). It is not surprising, then, that Human Cluster 1 genes, which show underexpression throughout the differentiation time course in mutant cells and show enrichment for cell adhesion genes (p = 2 × 10^−3^; Tables S4 and S5), are also enriched for ZEB1 DNA binding motifs (Fig. 4B; Table 1). In mouse cells, however, Zeb1 motifs were associated with the cluster of genes highly associated with cell–cell junctions and showing inappropriately high expression levels at all time points (Cluster 1: Fig. 5D and Table 1), consistent with aberrantly low expression of the Zeb1 repressor. While transgenic overexpression of ZEB1 was reported to increase neural differentiation of hESCs (Jiang et al., 2018), it did not lead to precocious differentiation of self-renewing hESCs, and hence is unlikely to be the principal factor behind the precocious differentiation seen in hiPSCs lacking MBD3.

It is possible that differences in transcriptional responses to differentiation in human and mouse cells could be due to the fact that, unlike mouse cells, human PSC are unable to maintain a stable self-renewing state in the absence of MBD3/NuRD, and are, in effect, responding to loss of self-renewal conditions when they have already started to differentiate. One possible, trivial explanation for this difference in the ability of mouse and human PSC to self-renew could be due to differences in the constituents of media used for self-renewal culture. Both mEpiSC culture and hiPSC culture rely on FGF2 and activation of SMAD2/3 through addition of Activin or TGFβ (Brons et al., 2007, Chen et al., 2011, Tesar et al., 2007), while naïve mouse ES cells are maintained through LIF signalling and dual inhibition of GSK3 and MEK/ERK (Ying et al., 2008). One consistent difference between mouse and human PSC culture media is the inclusion in human media of ascorbic acid (Vitamin C). Ascorbic acid has been shown to increase the activity of TET enzymes, which promote the demethylation of 5-methylCytosine in DNA, though this has been shown to promote a more naïve state, rather than promote differentiation (Blaschke et al., 2013, Yin et al., 2013). *Mbd3*-KO mESCs contain a reduced amount of DNA methylation relative to wild type cells (Latos et al., 2012), consistent with them being less able to differentiate. It is therefore unlikely that an increase in TET enzyme activity would be behind the precocious differentiation seen in hiPSC cultures. Rather, we suggest that the differences observed between human and mouse PSC in self-renewal or the ability to initiate an appropriate developmental response are most likely due to differences between the two species. As pointed out previously (Takashima et al., 2014), primates have not evolved the ability to undergo embryonic diapause (Nichols and Smith, 2012), and hence pluripotency may be a less stable state in humans than in mice, and consequently be less tolerant to the loss of a major chromatin remodelling complex such as MBD3/NuRD.

The mouse and human NuRD complexes present in primed PSC appear to be biochemically very similar, and our methods identified no notable species-specific interactors or alternate stoichiometries (Fig. 1). By chromatin immunoprecipitation, NuRD is found at all active enhancers and promoters in both mouse and human cells (Bornelöv et al., 2018, Burgold et al., 2019, de Dieuleveult et al., 2016, Gunther et al., 2013, Miller et al., 2016, Shimbo et al., 2013), but only a relatively small proportion of these genes changes expression after *MBD3* deletion (Figs. 4A, 5A) (Bornelöv et al., 2018). This is because NuRD acts to fine-tune expression through nucleosome mobilisation, and to cement longer-term gene expression changes through histone deacetylation activity (Bornelöv et al., 2018, Liang et al., 2017). NuRD’s fine-tuning function also works to ensure cells are able to respond appropriately when stem cells are induced to undergo lineage commitment. Yet the actual series of molecular events through which chromatin remodellers facilitate a cell’s ability to respond to differentiation cues remain ill-defined. The rapid development of single molecule and single cell analyses should allow us to now define exactly how chromatin remodellers, signalling molecules and transcription factors all interact at regulatory sequences to allow cells to respond quickly to changes in the local environment.

## 5 Conclusions

NuRD acts to prevent transcriptional ‘noise’ amongst genes that should be off in pluripotent cells, and it also modulates active transcription. In mouse cells the increased transcriptional noise in the absence of MBD3/NuRD is tolerated by the cells and they can stably self-renew, whereas human cells become destabilised by this noise and are unable to remain in a self-renewing state. When induced to differentiate the human cells will not be in a homogeneous state and, as a population, fail to induce the gene expression programmes necessary for successful lineage commitment. In contrast the mouse cells remain in a self-renewing state until induced to differentiate when, although they can initiate many of the correct gene expression programmes, fail both to maintain a differentiation trajectory and to commit to a specific lineage. We propose this difference arises due to a fundamental difference between mouse and human primed pluripotent cells.

## Declaration of Competing Interest

The authors declare that they have no known competing financial interests or personal relationships that could have appeared to influence the work reported in this paper.
