# Supplementary material for: Differential regulation of lineage commitment in human and mouse primed pluripotent stem cells by the nucleosome remodelling and deacetylation complex
Source: Stem Cell Res. 2020 Jul;46:101867. doi: 10.1016/j.scr.2020.101867 (PMC7347010; doi:10.1016/j.scr.2020.101867)
Supplement: Supplementary data 1 [file mmc1.docx]

**Figure S1. Human alleles used in this study.** A) Schematic of the human *MBD3* locus. Filled boxes represent coding exons, open boxes represent non-coding exons. B). Schematic of the *MBD3-FLAG* allele. A 3xFLAG epitope was knocked-in immediately upstream of the stop codon in Exon 6 on one *MBD3* allele. C). Schematic of the *MBD3*-KO alleles. Exons 2 and 3 were replaced by a Puromycin resistance cassette flanked by Rox sites. After the first round of targeting, the Puromycin cassette was removed by transient Dre expression, followed by a second round of targeting to mutate both *MBD3* alleles.

**Figure S2. Expression and GO-term plots for Clusters 5 and 6 from Figure 4A.** There were no significant (P.adj≤0.01) GO terms associated with Cluster 5 genes. Error bars indicate standard deviations of average expression.

**Figure S3. Failure of neural differentiation in mouse *Mbd3*-KO epiStem cells**. Wild type or *Mbd3*-KO cells after 8 days of neural differentiation were stained with antibodies indicated at top or DAPI. The final panels show merged images. Scale bars represent 100µm.

**Figure S4. Validation of gene expression changes in Mbd3-KO mouse EpiSC lines.** Gene expression during neural differentiation of two independent pairs of WT and *Mbd3*-KO EpiSC lines was measured by RNA-seq (Light blue and Magenta, top plots; as in Figure 5) or by qRT-PCR (red and blue lines, bottom plots) for indicated genes. The EpiSC lines were independently derived (see Methods) and differentiated at different times, but show the same changes in gene expression.

**Figure S5. Expression profiles of *ZEB1*, *CRB3* and *INADL* in hiPSCs and mEpiSCs.** A) RT-qPCR of *ZEB1,* *CRB3* and *INADL* in hiPSCs during neuroectodermal differentiation. The y-axis shows the relative expression and the x-axis shows the day of differentiation. The blue line represents the WT while the red line represents *MBD3*-KO hiPSCs (n=3 replicates/timepoint/condition). Error bars indicate the standard deviation. B) Expression profiles of *Zeb1*, *Crb3* and *Inadl* from mEpiSC RNA-seq data. The y-axis represents TPM (Transcript Per Million) while the x-axis and the different shapes represent the days of differentiation (n≥3 replicates/timepoint/condition). Blue indicate the WT and magenta represent Mbd3-KO mEpiSC.

**Figure S6. Genes misexpressed in human cells behave differently in mouse cells** A) Schematic of the analysis: the Venn diagram shows the overlap of differentially expressed genes identified in human cells, and the identified human orthologues of those identified in mEpiSCs. K-means clustering of this set of genes in human data led to the formation of three gene clusters. B). GO terms (“Functional annotation”; P.adj ≤ 0.01) and expression profiles are shown for the human gene clusters. Error bars indicate standard deviations of average expression. K-means clustering has been performed on the mouse gene orthologues of each human cluster resulting in 3 subclusters related to human cluster 1, 3 subclusters related to human cluster 2 and 4 subclusters related to human cluster 3. Heat maps of expression for orthologues in the mouse differentiation experiment are shown at right. There were no significant GO terms associated with Cluster 1 genes.

**Supplemental Tables**

**TableS1**. hiPSC_WT_vs_MBD3KO_day0_RNAseq_Analysis

**TableS2**. hiPSC_RNAseq_Analysis

**TableS3**. hiPSC_GOenrichment_Analysis

**TableS4**. hiPSC_Revigo

**TableS5**. mEpiSC_RNAseq_Analysis

**TableS6**. mEpiSC_GOenrichment_Analysis

**TableS7**. mEpiSC_Revigo

**TableS8**. hiPSC_Cluster_Genenames_Comparison_mEpiSCvshiPSC

**TableS9**. Gennames_GO_HumanMine_Revigo_Comparison_mEpiSC_hiPSC

**TableS10**. GO_Enrichments_HumanMine_Revigo_Comparison_mEpiSC_hiPSC

**TableS11**. Mouse_SubClusters_Related_to_Human_Clusters
